# How Random are Intrinsically Disordered Proteins? A Small Angle Scattering Perspective

**DOI:** 10.2174/138920312799277901

**Published:** 2012-02

**Authors:** Véronique Receveur-Bréchot, Dominique Durand

**Affiliations:** 1 IMR-CNRS – UPR3243, 31, Chemin Joseph Aiguier, 13402 Marseille Cedex 20, France; 2 IBBMC, CNRS UMR 8619, Université Paris-Sud, 91405 Orsay, France

**Keywords:** Denatured state, ensemble of conformations, induced folding, protein folding, random coil, small angle x-ray scattering, unstructured protein, wormlike chain.

## Abstract

While the crucial role of intrinsically disordered proteins (IDPs) in the cell cycle is now recognized, deciphering their molecular mode of action at the structural level still remains highly challenging and requires a combination of many biophysical approaches. Among them, small angle X-ray scattering (SAXS) has been extremely successful in the last decade and has become an indispensable technique for addressing many of the fundamental questions regarding the activities of IDPs. After introducing some experimental issues specific to IDPs and in relation to the latest technical developments, this article presents the interest of the theory of polymer physics to evaluate the flexibility of fully disordered proteins. The different strategies to obtain 3-dimensional models of IDPs, free in solution and associated in a complex, are then reviewed. Indeed, recent computational advances have made it possible to readily extract maximum information from the scattering curve with a special emphasis on highly flexible systems, such as multidomain proteins and IDPs. Furthermore, integrated computational approaches now enable the generation of ensembles of conformers to translate the unique flexible characteristics of IDPs by taking into consideration the constraints of more and more various complementary experiment. In particular, a combination of SAXS with high-resolution techniques, such as x-ray crystallography and NMR, allows us to provide reliable models and to gain unique structural insights about the protein over multiple structural scales. The latest neutron scattering experiments also promise new advances in the study of the conformational changes of macromolecules involving more complex systems.

## 1 INTRODUCTION

Intrinsically disordered proteins (IDPs) are currently in the limelight of the most recent and exciting structure-function relationship studies. These proteins have overthrown the long-lived idea that a definite 3D structure of a protein dictates its function [1,2]. Far from being the exception that proves the rule, they have revealed to be extremely abundant in the cell, especially in eukaryotes, and have been shown to fulfill numerous essential functions in the cellular cycle [3, 4]. Most of them participate in intricate interaction networks and are implicated in molecular recognition processes with multiple partners [5, 6], sometimes through an induced folding mechanism, in which the disordered protein gains secondary structural elements upon binding to its partner [7]. Because these proteins have been shown to be at the crossroads of many disease-related signaling pathways, they are considered a rich and unexplored reservoir of original targets for new drug design strategies based on protein-protein interactions [8-10]. Therefore, while understanding the molecular background of their function is crucial, linking their structural properties to their function is still very challenging because of the lack of rigid regular structure. Attempting to elucidate their structure-function specificities is extremely difficult, sometimes impossible, using a single classical structural method, such as x-ray diffraction or Nuclear Magnetic Resonance (NMR) [11]. Only a strategic combination of complementary structural and biophysical techniques would allow one to decipher their mode of action at the structural level [12]. Among these techniques, small angle x-ray and neutron scattering (SAXS and SANS) have become increasingly valuable and effective and are particularly well adapted to the study of such proteins. The recent extraordinary success of small angle scattering techniques has been possible due to the growing number of programs and algorithms that have been developed in recent years that exploit all the structural information contained in the data on the conformation of proteins [13, 14]. Furthermore, especially in the case of proteins containing disordered domains or bound to a structured partner, small angle scattering techniques are extremely powerful when used in combination with other methods, in particular with high-resolution methods, such as NMR or x-ray crystallography.

SAXS had long been used in protein folding studies, benefiting from earlier studies in polymer chemistry [15]. In particular, it was one of the very few techniques that could characterize the denatured state of proteins, which represents the other half of the folding equation [16, 17], and the identified folding intermediates, in particular the molten and the premolten globules [18]. It is therefore logical that some of the pioneering studies on IDPs used SAXS to demonstrate the disordered nature of a native protein [19, 20]. SAXS is also the method of choice for the study of proteins that cannot crystallize, which is typically the case for IDPs [21, 22]. It is currently the only available structural method for the study of large flexible proteins [13, 14, 23].

The term IDP actually covers a wide variety of objects, as pointed out by Dyson & Wright [24], ranging from fully disordered proteins, similar to random polymers, to multi-domain proteins containing only long or short disordered regions along with very well ordered domains, and even to molten globules that possess all or most of their secondary structure. As mentioned above, many intrinsically disordered regions may also be involved in molecular recognition and in the formation of macromolecular complexes. The strategy of how to analyze SAXS data is chosen according to the type of object under study, the biological questions being addressed, and the kind of complementary structural and biophysical data that are also available. In particular, SAXS allows one to (i) decipher the molecular dimensions of a protein in solution, (ii) infer the low-resolution shape of a protein or complex in solution, (iii) determine the structural arrangement of multidomain proteins, and (iv) assess the flexibility of a polypeptide chain through the distribution of conformations it can attain. When combined with high-resolution methods, the structural features of the protein or complex may then be described with more detail, allowing a deeper understanding of its molecular characteristics. Furthermore, the most recent advances in computational analyses now make it possible to take full advantage of combining SAXS with incredibly numerous techniques, especially through the wide variety of information provided by NMR, and to generate large ensembles of meaningful configurations [25-32]. Consequently, SAXS is now able to provide a comprehensive picture of the dynamic behavior of IDPs and their structural properties both free in solution and bound to a partner. The most recent successes using SAXS attest to its ability to answer real biological questions related to these enigmatic and fascinating disordered proteins and their functions. SAXS therefore will be at the forefront of the forthcoming structural studies of IDPs. 

In the present paper, we review the various strategies that can be employed to decipher the structural and dynamic features of IDPs using SAXS. The limits and pitfalls of data acquisition, data analysis, and interpretation of results will also be underlined, as SAXS is an inherently underdetermined method confronted to an ill-posed problem, and therefore has few safeguards. Finally, the latest advances in the technique, especially in combination with cutting-edge computational and biophysical methods, will be presented, which will exemplify the fundamental questions regarding IDPs these studies could valuably address.

## 2 PRACTICAL EXPERIMENTAL ISSUES

A SAXS experiment measures the scattering intensity *I*(*q*) upon variation of the scattering angle 2*θ* as a function of the scattering vector *q* defined by $q=\frac{4\pi \sin\theta }{\lambda }$ , where λ is the wavelength of the radiation. According to Bragg’s law, the corresponding distance in real space of the scattering vector *q* is given by *d*=2π/*q*. This scattering curve *I*(*q*) hence contains information in the reciprocal space on the structure of the object in solution at different distance scales typically ranging from ~10 to several hundred Angstroms. The maximum size of the object that can be studied by SAXS is only limited by the smallest angles, or scattering vector *q*, that the instrument can attain for measuring the scattering intensity. This minimum scattering vector, *q*, in reciprocal space or the corresponding maximum measurable distance, in real space, is therefore the most important parameter of a SAXS experiment as it determines the maximum size of the object that can be studied. Similar to crystallography, the resolution of the experiment is defined by the smallest distance that can be attained and therefore by the maximum value of the scattering vector q_max_ of the experiment. Typically, a synchrotron SAXS experiment allows a resolution of a dozen of Angstroms, *i.e.*, q_max_ ≈0.5 Å^-1^. However, contrary to X-ray diffraction, which defines the resolution by the minimum distance that can be resolved between two separated objects, the notion of resolution in SAXS is more vague because of the orientational averaging of the proteins in solution and because of the absence of any parameter, such as I/sigma(I) in crystallography, that would assess the signal-to-noise ratio. The maximum scattering vector *q* therefore does not yield the smallest distance allowing for the separation of two distinct objects but only a distance below which details provided by modeling are not significant. Noteworthy efforts are underway to extend the SAXS limits up to q ≈2 Å^-1^, *i.e*., distances of 3-4 Å, by covering the wide angle X-ray scattering (WAXS) domain to better characterize the solvent surrounding the protein [33] and to follow subtle conformational changes upon, for example, ligand binding [34-36]. 

The measured scattering profile at very low angles is highly sensitive to the presence of large objects, especially aggregates. Because this part of the scattering curve contains information on the dimensions of the protein, it is therefore crucial to be able to discriminate between the effect of aggregates and the effect of a wide variety of extended and collapsed conformations adopted by the IDP on the scattering curve. The presence of aggregates in solution will translate into an increase in the scattering intensity at low *q*, preventing an accurate measurement of the radius of gyration and maximum diameters. This issue is particularly crucial for IDPs because they possess large dimensions and the zone of application of Guinier law is reduced (see below). The experimental accessible *q*-range allowing one to determine the molecular dimensions of IDPs is therefore often very small. Consequently, highly monodispersed samples with no trace of aggregation are absolutely required. A solution may be provided by a size-exclusion FPLC or HPLC device connected upstream to the measurement capillary that separates the aggregates from the protein online. Such a set-up is now being proposed on several beamlines, such as at the SWING at SOLEIL synchrotron, near Paris, France [37], at the BL-10C station at the Photon Factory, in Tsukuba, Japan [38, 39], and the SAXS/WAXS beamline of the Australian synchrotron in Melbourne, Australia [40].

Another concern is the effect of intermolecular interactions in non-ideal solutions on the measured scattering intensity at low angles. The experimental scattering intensity is expressed as: I(q)=F(q). S(q), where F(q) is the form factor of the scattering object, which contains all the information on the shape of the protein, and S(q) is the structure factor, which is related to interparticle interactions. The structure factor is equal to 1, S(q)=1, in the case of an ideal solution without any intermolecular interactions and tends toward 1 only at medium and large* q* values in the case of real solutions. The scattering spectrum at low q is thus artificially decreased or increased in the presence of repulsive or attractive interactions, respectively. Measurements at different protein concentrations and extrapolation to zero concentration are therefore often required to eliminate the contribution of the structure factor on the measured scattering intensity at low angles. 

In any case, a careful inspection of the experimental value of the forward intensity I(0) related to the molecular weight M_w_ of the protein (eq. 1) is required to detect the presence of aggregates or intermolecular interactions.

(1) $$I(0)=\frac{cM_{w}}{N}{\left[\left(\rho_{p}-\rho_{s}\right)v_{p}\right]}^{2},$$

where *c* is the protein concentration in solution, N is Avogadro’s number, ρ_p_ and ρ_s_ are the scattering length density of the protein and of the solvent, respectively, and v_p_ is the specific volume of the protein. SAXS is one of the very few methods that can directly determine the molecular weight of a macromolecule. In contrast to dynamic light scattering or size exclusion chromatography, for example, the measurement does not rely on the hydrodynamic properties of the macromolecule or on any assumption about the shape of the protein. An accurate determination of the molecular weight depends strongly on (i) the accuracy of the I(0) determination through the Guinier or the Debye law (see below) and is therefore very sensitive to the presence of aggregates in the solution and intermolecular interactions; (ii) the calibration of the measured data into the absolute scale (cm^-1^); calibration using pure water is preferable compared to a standard protein whose concentration and specific volume will not be determined as precisely, because the scattering intensity of water is precisely tabulated; (iii) the accuracy of the measurement of the protein concentration, which requires good knowledge of the extinction coefficient of the protein, while IDPs are often depleted of tryptophans; and (iv) the calculation of the specific volume v_p_ of the protein using, for example, the NucProt program [41], SEDNTERP (http://www.jphilo.mailway.com/default.htm and [42]) or other tables [43]; notably, unstructured proteins tend to have a lower specific volume than globular folded proteins, which often display pockets [44], giving rise to slightly lower I(0).

Typical scattering curves of IDPs are characterized by the absence of any specific feature, contrary to globular objects [45]. IDPs indeed exhibit many different conformations, which all display a different scattering profile. The resulting scattering curve is a combination of these numerous contributions and is therefore considerably smoothened upon averaging. Because of the absence of marked specific features on the scattering curve of IDPs, it is essential to collect data of the highest possible quality with good statistics, even at large *q* values, and accurate error bars because this plays a crucial role in the accurate determination of the distance distribution function and in the subsequent data analysis. The reader can refer to a recent review from Jacques and Trewhella [46] that provides a very useful set of guidelines for the ‘good laboratory practice’ of a scattering experiment and for the critical evaluation of scattering data. 

Finally, considering the concentrations required for SAXS (mg/mL of protein in the beam) for a sufficient signal-to-noise ratio allowing buffer subtraction and according to the law of mass action, reliable SAXS experiments performed on complexes involving several partners (protein, DNA, and others) require that the complex is of high affinity with dissociation constants (Kd) below the µM range. This is an essential prerequisite to avoid measuring the signal arising from an undefined mixture of the isolated partners in equilibrium with the complex. A solution can again be provided by the use of an HPLC column upstream of the measurement capillary, provided that the complex is stable enough to not dissociate completely upon elution of the column.

## 3 HOW TO EVALUATE AN IDP BY SAXS 

The first characteristics that are usually inferred from scattering data without requiring any modeling are (i) the radius of gyration, (ii) the distance distribution function P(r), which is the histogram of the distances within a protein, and (iii) the scattering profile in a Kratky plot (q^2^I(q) vs. q), which directly reports on the compact or unstructured nature of the polypeptide chain. 

### 3.1 The Radius of Gyration

The radius of gyration is the first parameter yielded by SAXS and provides information on the average size of the scattering object in solution. Because IDPs are prone to adopt large extended conformations, the radius of gyration is a particularly relevant parameter to evaluate an IDP using SAXS. The radius of gyration, *R_g_,* is defined by the root mean square of the radii, *r,* in the volume, v, of the protein and is given by the following equation:

(2) $$R_{g}^{2}=\frac{\int_{v}\left(\rho \left(\vec{r}\right)-\rho_{s}\right)r^{2}d^{3}r}{\int_{v}\left(\rho \left(\vec{r}\right)-\rho_{s}\right)d^{3}r},$$

where ρ(r) is the scattering length density. The radius of gyration is generally determined using the Guinier Law:

(3) $$I\left(q\right)=I\left(0\right)\exp\left(-\frac{q^{2}R_{g}^{2}}{3}\right)\cdot$$

The Guinier law can theoretically be applied to any particle whatever its shape. The radius of gyration is inferred from the slope of the Guinier plot, which represents the logarithm of the scattering intensity versus *q*^2^*. *A Guinier plot is only linear over a restricted region of the scattering spectrum: *q*R_g_ < 1.0. This region may sometimes be extended to *q*R_g_ < 1.3 for well-folded proteins, but for an IDP, the region is actually reduced to *q*R_g_<0.8 and is sometimes even smaller [13, 47] because of the multiple sizes adopted by an IDP. Therefore, there may only be a few usable points in the experimental spectrum, thus limiting the accuracy of the measured R_g_. The Debye law offers an interesting alternative to determine the radius of gyration of fully denatured or very disordered proteins. It describes the behavior of a Gaussian chain and can be applied to a wider region of the scattering spectrum, up to *q*R_g_ < 3, for a polymeric random coil [48], although a narrower q-range, up to q < 1.4, provides a much more reliable R_g_ for unstructured proteins [49]. The Debye law is given by the following expression:

(4) $$\frac{I\left(q\right)}{I\left(0\right)}=\frac{2}{x^{2}}\left(x-1-e^{-x}\right),$$

where *x* = (*qR_g_*)^2^.

The experimental radius of gyration of an IDP can be compared to theoretical or experimental values published for a globular and an unfolded protein of the same number of residues to quantitatively assess the extended nature of the IDP and to estimate whether the protein behaves as a random coil or whether it is more compact due to putative residual structure. Random coils usually refer to highly unfolded or disordered proteins with no or almost no structural elements with the vast majority of residues solvent exposed.

Several systematic studies based on Flory’s theory of polymer physics [48] may be used as references to determine how far the IDP differs from a random coil. Flory showed that the radius of gyration follows a power law, $R_{g}=R_{0}N^{v}$ , where N is the number of monomers of the polymer, R_0_ is a constant, and ν depends on the structural behavior of the polymer chain in the solvent. Thus, the theoretical value of ν for a spherical compact globule is 1/3, whereas the predicted value of ν is 0.5 for a random coil and 0.588 for a polymer in “good solvent” or an excluded volume chain [48]. In an excluded volume chain, the interactions are dominated by steric repulsions between the monomers of the chain, as in the case of the amino acids in a polypeptide chain in the presence of strong denaturant concentrations. Plaxco and co-workers compiled results in the literature on a wide range of native globular proteins and on strongly denatured proteins [50-52]. They discovered that the experimentally-derived radii of gyration follow the scale law as a function of the number, N, of residues of the protein with ν = 0.38 ± 0.05 for native globular proteins [52], which is close to the 1/3 predicted for a sphere, and ν = 0.598 ± 0.028 with R_0_=1.93 for completely denatured proteins, which is close to the expected value of 0.588 for excluded volume chains [48]. Wilkins *et al.* obtained similar results on a smaller subset of native and denatured proteins, with R_0 _= √(3/5) x 4.75 and ν = 0.29 for native proteins and with R_0 _= 2.23 and ν = 0.58 for proteins under strongly denaturing conditions [53]. In the case of an IDP in an aqueous buffer, Bernado and Blackledge infer lower values of R_g_, with R_0 _= 2.54, and ν = 0.522, which is closer to the expected exponent for a random coil [54]. They obtained this result by calculating the scattering intensity of the ensemble of conformations adopted by polypeptide chains of N residues. This result was then consistent with a small set of experimentally derived R_g_ values of IDPs. However, it is recognized now that there probably exists a continuum between ordered and fully disordered proteins, resulting from a wide diversity of sequences. These predictions of R_g_ from the number of residues of IDPs therefore constitute lower and upper bounds, and significant differences from the upper bound actually reveal global or local structural restraints, indicating how the IDP deviates from the random coil. Indeed, Plaxco and co-workers explored the effect of local or residual structures on the scaling behavior and dimensions of unstructured proteins and showed that residual helical structures contract the protein, whereas PPII helices tend to increase the dimensions of the protein beyond the value expected for a random coil [55].

Finally, the radius of gyration of a protein can be compared to the hydrodynamic radius, R_h_, determined by DLS or pulse-field gradient NMR (PFG-NMR). The hydrodynamic radius, or Stokes radius, is the radius of the equivalent sphere that diffuses with the same diffusion coefficient. The R_g_/R_h_ ratio is (3/5)^1/2^ for a globular protein and approximately 1.4 for a denatured protein. Although not very informative, any intermediate value of this ratio ascertains the presence of more or less residual structure (*molten globule* and *premolten globule*). This was the approach of Uversky and coll., for example, who reported on the intrinsically disordered C-terminal domain of caldesmon [56] and on α-, β- and γ-synucleins [57]. 

### 3.2 The Distance Distribution Function P(r)

The other dimensions of the protein can be accessed through the distance distribution function P(r), which is inferred by the Fourier transform of the scattering intensity F(*I*(*q*)) using the programs GNOM [58] or GIFT [59]:

(5) $$P\left(r\right)=F\left(I\left(q\right)\right)=\int I\left(q\right)e^{-i\vec{q}.\vec{r}}\mathrm{dq}.$$

The P(r) function is a histogram of all the interatomic distances, *r, *within the protein. The maximal value of *r* for which *P*(*r*) is not equal to zero, D_max_, corresponds to the maximum diameter of the protein. This histogram and the value of D_max_ contain valuable information on the shape, the anisotropy, and the degree of compactness of the protein. 

Typical P(r) functions of IDPs are very asymmetric compared to the highly symmetric P(r) function of globular proteins that lack any marked features or breaks, and end with a smooth concave curvature. They often display an extended tail due to the variety of extended conformations present in solution. The presence of aggregation in solution also translates into a similar extended tail. The complete absence of aggregates in solution is therefore absolutely necessary in the case of IDPs to avoid misinterpreting the data. The P(r) functions of proteins containing several globular domains tethered by long disordered regions are characterized by peaks at low r values, corresponding to the intradomain distances, and a tail with more or less pronounced shoulders corresponding to the interdomain distances and depending on the flexibility of the linker (Fig. (**1**)). 

The radius of gyration and forward intensity I(0) can also be inferred from the P(r) function according to equation (6).

(6) $$R_{g}^{2}=\frac{\int_{0}^{D_{\max}}r^{2}P\left(r\right)\mathrm{dr}}{2\int_{0}^{D_{\max}}P\left(r\right)\mathrm{dr}}\mathrm{and}I\left(0\right)=4\pi \int_{0}^{D_{\max}}P\left(r\right)\mathrm{dr}$$

This alternative method to determine the radius of gyration and I(0) is interesting because it does not rely on any model (Debye or Guinier) and uses the entire scattering spectrum. The R_g_ inferred from this equation often yields slightly larger values than with the Guinier law [45] mainly because the Guinier law is less appropriate to describe an unfolded chain and often underestimates the radius of gyration of extended chains. It is therefore always interesting to compare the values of R_g_ obtained from these two methods. Finally, determining I(0) through the P(r) function allows one to cross-check the values obtained by the different methods and to ascertain the quality of the data.

It is worth noting that it may be interesting to confront the values of R_g_ and D_max_ of an IDP. Whereas the radius of gyration is an average dimension of all the conformers in solution, the maximum diameter D_max_ is inferred from the most extended conformations significantly present in solution. Thus the flexibility of linkers in bimodular cellulases could be assessed by comparing these dimensions for different variants [60]. Cellulase Cel45 is composed of a catalytic domain and a small cellulose-binding domain whose structures have been solved. The dimensions of the full-length protein allowed a direct inference of the maximum distance of the linker within the protein and demonstrated that the linker was very extended. In a variant of Cel45 in which two amino acids of the linker were replaced by two prolines resulting in a stretch of five consecutive proline residues, the maximum dimensions were the same as in the wild-type protein, whereas the radius of gyration, and thus the average dimensions of the variant with the polyproline stretch, were larger than those of the wild-type protein. The marked bimodal distance distribution function of the variant compared to the smoother shoulder observed in the P(r) function of the wild type cellulase (Fig. (**1**)) also indicated that the most extended conformations were more abundant in the variant, whereas the wild-type protein was more flexible and could adopt both compact and extended conformations. The profile of the distance distribution function and the dimensions that it provides therefore reveals much information on the compactness, anisotropy, and flexibility of a protein. Clearly, only a thorough analysis of the scattering curve by using an ensemble of conformations (see below) provides quantitative information on the flexibility and distribution of conformations that the protein may adopt. Nevertheless, examining the P(r) function, D_max_, and R_g_ provides rapid information on the nature of the linkers and on the different subpopulations without any assumptions and thus guides the selection of a strategy for the further analysis of the scattering curves. 

### 3.3 The Kratky Plot

The Kratky plot is an extremely useful representation of the scattering intensity to quickly assess the globular nature of a polypeptide chain without any modeling. The Kratky plot plots the scattering pattern as *q*^2^*I*(*q*) versus *q*. The scattering intensity *I*(*q*) of a globular protein with a well-defined, solvent-accessible surface follows the Porod law and decreases as *q*^-4^ in the large *q *region. As a result, the corresponding Kratky plot exhibits a typical bell-shape with a well-defined maximum. Conversely, for a random chain, the scattering intensity has a limiting behavior of *q*^-2^ at high *q*, as indicated by the Debye law (Eq. 4). Therefore, the Kratky plot of a fully unfolded protein will exhibit a plateau in this *q *region, sometimes followed by an increase as *q* increases, depending on the local rigidity of the chain. Nevertheless, this representation is not able to distinguish between fully folded and partially unfolded proteins containing structured regions of significant size, which also results in bell-shaped Kratky plots. To obviate this problem, Perez and co-workers highly recommend plotting a dimensionless Kratky plot [61], as is commonly done in other fields, such as polymer science. In this dimensionless Kratky plot, the intensity *I*(*q*) is normalized to the forward scattering intensity *I*(0), and *q *is normalized to the radius of gyration of the protein. Multiplying *q* by the radius of gyration makes the angular scale independent of protein size, while I(*q*) divided by I(0) becomes independent of the molecular weight of the protein as I(0) is proportional to the molecular weight (Eq. 1). This normalization allows one to compare Kratky plots of globular and extended proteins, whatever their size, and thereby to infer the maximum amount of information from this representation. The scattering pattern of a globular protein in a normalized Kratky plot exhibits a maximum value of 1.104 for qR_g_=√3, whatever the size of the protein. Conversely, for a random chain, the curve rises with increasing angle to reach a nearly flat region at a value between 1.5 and 2 followed at high *q* values (typically *q* > 0.2-0.3 Å^-1^) by a further increase depending on the rigidity of the polypeptide chain. Dimensionless Kratky plots of partly disordered proteins display distinctive intermediate profiles between the two extremes (Fig. (**2**)).

## 4 ASSESSING THE FLEXIBILITY OF FULLY DISORDERED PROTEINS WITH THE THEORY OF POLYMER PHYSICS

Some IDPs are disordered along their entire sequence, whereas other so-called IDPs actually contain one or several long intrinsically disordered regions (IDRs) separated by globular domains with a definite function. If these IDRs remain active when isolated from the rest of the protein, they constitute individual domains, and their structural and function properties are often individually studied. Comparing the radii of gyration of these fully disordered proteins or domains with the expected R_g_ yielded by the empirical power law described above can provide information on the degree of structural disorder in the protein. However because it is a macroscopic parameter, the radius of gyration is not sufficiently sensitive to detect slight conformational restraints. Analyzing the entire scattering curve represents a step forward to infer and utilize all the quantitative information contained in the scattering spectrum. 

The theory of polymer solutions can be used to describe the behavior of highly unfolded or disordered polypeptide chains in solution with the worm-like chain model (WLC, also referred to as the Kratky-Porod chain model) [62]. The worm-like chain is a model chain with a persistence length that takes into account the local rigidity of the polypeptide chain. This rigidity accounts for the range of possible torsion angles between two adjacent residues and also for putative residual structure. Two parameters describe this Kratky-Porod chain: the contour length, *L*, and the statistic length or Kuhn length, *b*, which is twice the persistence length. The persistence length is a measure of the stiffness of the polypeptide and is defined as the length over which the polymer naturally stays straight. A higher persistence length indicates higher rigidity. This rigidity may be due to excluded volume interactions in the case of proteins denatured by chaotropic osmolytes, such as urea or guanidinium chloride, or to the presence of structural elements in the case of intrinsically disordered proteins in aqueous solutions. The contour length, *L, *is the length of the linearly extended chain without stretching the backbone. The scattering intensity follows this expression:

(7) $$\frac{I\left(q\right)}{I\left(0\right)}=\frac{2}{x^{2}}\left(x-1+e^{-x}\right)+\frac{b}{L}\left[\frac{4}{15}+\frac{7}{15x}-\left(\frac{11}{15}+\frac{7}{15x}\right)e^{-x}\right],$$

with *x* = *q*^2^*Lb*/6. This formula is valid for *L*/*b* >10, which means that the chain is long enough compared to the statistic length, and for *q*<3/*b*. For a completely unfolded or disordered protein, such as a random coil, the value of the statistical length *b* is expected to be ~18-20 Å [47]. Similarly, the theoretical contour length of a random coil is equal to N*l_0_f*, where N is the number of residues, *l_0_* is the distance between two C_α_ (*l_0_*=3.78Å), and *f* is a geometrical factor that arises from the fact that an unfolded chain is not linear but zigzags and is equal to 0.95. A smaller contour length reveals the presence of local structures. Finally, the radius of gyration of a random coil can also be inferred from the values obtained for *L* and *b* according to the following relationship:

(8) $$R_{g}=b{\left[\frac{y}{6}-\frac{1}{4}+\frac{1}{4y}-\frac{1}{8y^{2}}\right]}^{1/2},$$

where *y*=*L*/*b.* Such analyses using the Kratky-Porod model were first performed on completely denatured globular proteins. It was thus shown that CheY denatured by 5 M guanidinium chloride displays a significant rigidity all along the polypeptide chain due to the excluded volume generated by solvation of the chain by the denaturing osmolyte. *b *was equal to 28 Å, and *L* was lower than the value expected for a random coil [63]. In contrast, heat-denatured neocarzinostatin (NCS) exhibits values of *L* and *b* close to those of a random coil [49]. These results can be compared to those obtained for IDPs. For example, the radius of gyration of the intrinsically disordered XPC is slightly lower than that expected for a random coil according to equation (8), which is consistent with the existence of short elements of secondary structure observed by circular dichroism [64]. Conversely the R_g_ of disordered PIR domains better corresponds to a random coil [47]. Similarly, the contour length of Msh6-NTR, of 1078 Å, compared to 1091 Å for a random coil, and its statistical length of 18.7 Å, which yields a persistence length of 9.35 Å, corresponding to roughly three amino acids, are consistent with a polypeptide chain adopting random conformations. The case of the proline-rich salivary proteins IB5 and II-1ng is more subtle than the previous examples [65]. The radius of gyration and the maximum diameter of these proteins are larger than those expected for a random coil, indicating that these proteins have strongly extended conformations. Conversely, the statistical lengths of these two proteins are of 29.7 and 29.9 Å, respectively, revealing the existence of secondary structure elements. Similarly, their contour lengths, *L,* are significantly lower than that expected for a random coil, of 188 and 364 Å, instead of the theoretical values of 251 and 503 Å for IB5 and II-1ng, respectively, which is also consistent with secondary structure elements such as short PPII or PPI helical fragments. The high R_g_ value together with the higher statistical length and lower contour length reveal that these proteins are more extended than a classical random coil because of PPI or PPII helical fragments that stretch the polypeptide chain. These examples illustrate that many insights on the structural restraints in an IDP can be gained by analyzing the scattering curve with the theory of polymer physics.

## 5 3D MODELING OF IDPS USING SAXS

The incredibly growing success of SAXS in the past few years has arisen from the latest advances in SAXS computational data analysis and the possibility to yield more and more detailed 3D models of the macromolecule under study, even for IDPs. SAXS thereby became extremely powerful and could provide highly important clues on the structural and functional mechanisms of flexible systems with crucial biological roles, including IDPs [23]. However, SAXS is confronted with the ill-posed problem of inferring a 3D structure from a 1D scattering curve, leading to the crucial question of the uniqueness of the solution, as has been addressed by Svergun and co-workers [66]. Consequently, the theoretical scattering curve of several different models may fit the experimental data with the same adequacy. This issue is even more acute for IDPs, which already exist as ensemble conformations in solution. The strategy to solve this ambiguity and to infer reliable models is to impose constraints on the reconstructions, implemented as much as possible by adding external information from complementary techniques. SAXS can therefore provide more detailed structural insights on IDPs when complemented by other structural techniques, especially NMR or X-ray crystallography, which provide the high-resolution information missing from the SAXS data. Hence, 3D-models that gather the information provided by SAXS and by these techniques, are tremendously helpful for characterizing IDPs, either containing structured domains, or in complex with a structured partner, or containing residual structures described by high-resolution techniques, such as NMR. 

### 5.1 Overall Shape of a Protein or Complex

The development of new programs that restore the envelope of a scattering object from its scattering curve *ab initio* triggered the expansion of SAXS in structural biology a decade ago. Several programs that use different algorithms and apply different restraints for the calculation to converge faster are now available. All of these programs calculate the overall external shape of a protein by filling the volume of a bead model with beads of variable size and number. DAILA_GA, for example, uses a genetic algorithm [67], whereas SAXS3D uses a Monte Carlo-type reconstruction algorithm [68], and the program suite DAMMIN/ DAMMIF/MONSA/GASBOR uses a simulated annealing procedure [69, 70]. A comparison of these programs reveals that they are all able to properly retrieve the overall shape of a well-folded protein with a similar quality of fit at high resolution [71]. A considerable effort has been made to significantly decrease the calculation time in particular for DAMMIF with respect to DAMMIN [72]. Restraints applied in DAMMIN/DAMMIF [69] aim to minimize the interfacial area between the protein envelope and the solvent, imposing compactness and connectivity constraints, which may not be appropriate for proteins with a significant amount of intrinsic structural disorder. DAMMIN/DAMMIF also fits the data up to a resolution of 25 Å (q≈0.25 Å^-1^) [73]. GASBOR [70] uses the entire scattering curve up to a resolution of ~10 Å to generate a bead model in which each bead corresponds to a dummy residue (spheres of 3.8 Å diameter), the number of residues are equal to the number in the protein (with an upper limit of ~1800 dummy residues), and nearest-neighbor distribution constraints are applied. MONSA allows describing complex objects composed of several domains of different electronic densities or of different scattering lengths and is therefore very useful for protein/DNA or protein/RNA complexes, for example, or for small angle neutron scattering (SANS). 

Trying to retrieve the overall shape of a highly dynamic macromolecule such as an intrinsically disordered protein may at first glance appear meaningless. This shape provides at least a visual insight and confirms the parameters (R_g_, D_max_, contour and persistence lengths) inferred from the scattering curve already provided numerically, especially for entirely disordered proteins. The primary interest of the shape calculation is actually for objects containing both globular and disordered regions. This is the case for plurimodular proteins, in which linkers, or long disordered regions, tether globular domains as well as for complexes between an IDP and a globular folded partner. This strategy often allows one to locate the respective position of each globular domain whose atomic structure was already known either by X-ray diffraction, NMR or molecular modeling. Information on the compactness or the degree of disorder of the linker or predicted disordered region in between can then be inferred from possible protruding regions of the shape or from dimensions inside the complex between the different folded domains. 

Because of the inherent dynamics of these objects, the calculated shape is only a rough average of the global structure of the object in solution [45]. Interestingly, whereas for a globular rigid protein, the *ab initio *shape restoration is usually robust upon numerous calculation runs, the shape reconstructions of an IDP or of a highly flexible region may vary dramatically from run to run [47, 74-76]. Therefore, for a highly flexible object, after repeating the calculations of the restored shape to check the reproducibility of the yielded solution, it is essential to display the most typical shape among those obtained by each calculation. Averaging all these shapes would smooth all significant and informative features of the shapes, which slightly differ in size and location from one shape to another, and all of the relevant and significant information provided by a single shape would be lost. However, the program DAMAVER is extremely useful for rigorously selecting the most typical reconstruction. This program aligns the different shapes and calculates a normalized spatial discrepancy (NSD) between them. An NSD value below 0.7 for DAMMIN reconstructions and below 1.1 for GASBOR reconstructions indicates that the solution is stable. Significant outliers are discarded by the program, and the reconstruction with the lowest NSD is selected [66].

The use of shape calculation for proteins or complexes containing disordered regions can be illustrated by the study of the formation of cellulosomes, that constitute extremely active multienzymatic cellulolytic complexes [77]. The global shape of a complex along with the distances measured between the folded subdomains upon assembly of a minicellulosome revealed an unexpected compaction of the linker separating the cellulase domain and the dockerin domain upon binding of the dockerin domain to the cohesin domain. These data revealed a novel mechanism of remote induced folding of a disordered region several Angstroms from the binding site [77]. 

Another interesting aspect of shape calculation is when the crystal structure of the complex between the folded partner and the molecular recognition element of the IDP has been solved. Determining the overall shape of the complex is then tremendously useful for investigating the putative structure of the region of the IDP not directly involved in the binding interface. A pioneering example was provided by the SAXS structure of the complex between the full-length measles virus N_TAIL_ of the nucleoprotein in complex with the X domain (XD) of the phosphoprotein [78]. A reproducible and very recognizable bulky part of the *ab initio* restored shape of the complex could accommodate the atomic structure of XD associated with the 20-residue long alpha-MORE of N_TAIL_. The rest of the shape was highly variable from one run to another but always exhibited a long protuberance with varying bends and cross-sections. These data revealed that the 90 N-terminal residues of the protein remained disordered upon binding to XD (Fig. (**3**)). Another example is provided by the complex between the disordered translational repressor eIF4E binding protein 4E-BP and the initiation factor eIF4E [76]. Determining the shape of the isolated proteins and of the two proteins in complex revealed that 4E-BP wraps around eIF4E to form a fuzzy complex. These structures shed light on the mechanisms of regulation of eIF4E by the disordered 4E-BP, which involves other regions of the protein that were already suspected based on former NMR studies (Fig. (**4**)). The overall shape of the ternary complex composed of the full-length intrinsically disordered p27, the cyclin dependent kinase cdk2 and cyclin A could also provide insights into the mechanisms of inhibition of Cdk2/ cyclin A by p27 to limit cell proliferation [79]. The low-resolution envelope of the ternary complex obtained using SAXS displayed a large roughly spherical bulge on which the crystal structure of the complex composed of the N-terminal KID domain of p27, Cdk2 and cyclin A could be superimposed and a protruding elongated region that could accommodate an ensemble of models of C-terminal p27 obtained by molecular dynamics simulations. This structural organization indicated that the C-terminus of p27 remains highly flexible and is able to fold back onto the active site of Cdk2, where it could be phosphorylated and trigger a signaling cascade for degradation and cell division. 

### 5.2 Conformation of Disordered Regions within Proteins or Complexes

The very few previous examples selected from the growing number of such studies in the literature show to what extent a low-resolution 3D envelope may be sufficient to provide crucial information on the overall organization of IDPs in a complex. Nevertheless, the most recent advances in SAXS make it possible to go a step forward and to infer a molecular model of an isolated protein containing long disordered regions, such as multidomain proteins, or of a complex involving an IDP. These 3D-models can then provide essential clues on the mechanisms of molecular recognition of IDPs, especially when these models are obtained by combining results from other biophysical and structural methods that provide high-resolution information.

Several programs have been developed to restore the conformation of polypeptide chains in the disordered region of a protein amidst the more structured regions for which the atomic coordinates are known. The program BUNCH [80] combines a rigid body with an *ab initio* modeling approach. The folded domains with known structure are considered as rigid bodies, whereas the unstructured regions are modeled by chains of dummy residues. Their optimal positions and orientations are then calculated using a simulated annealing algorithm to fit the scattering data with restraints minimizing steric clashes and discontinuities in the chains. BUNCH is particularly well adapted to multidomain proteins with disordered regions between the globular domains. An extension of BUNCH, CORAL (COmplexes with RAndom Loops), is now available, which performs the same modeling but for complexes composed of several partners. If known, distance restraints between residues, such as the interacting residues, can be added. As for all the other programs developed by Svergun’s group, BUNCH and CORAL are easily available at http://www.embl-hamburg.de/biosaxs/.

The program DADIMODO has also been developed for proteins or complexes containing both structured and disordered domains [81]. It is based on a genetic algorithm and has been designed to combine SAXS and NMR data. Distance restraints, such as those provided by chemical shift mapping, and orientational restraints, provided by RDCs, may be added to the algorithm [82]. Unlike BUNCH, the program can also deal with very extended particles because it is not limited by the size of the complex or the number of harmonics (see below). Another advantage of this program is that it builds models using real amino acids and can therefore apply an energy minimization on the selected conformations. Finally, because it is open source, it is possible to insert potentials from other methods based on the user’s needs. Thus, by combining SAXS and NMR data using DADIMODO, Aliprandi *et al. *could describe the spatial organization and interactions between different subdomains of the ribosomal protein S1, thus shedding light on the structural events occurring during RNA binding [83]. 

The extraordinary interest in these approaches can be best exemplified by the quaternary structure of full-length p53, which was determined using SAXS coupled with the crystal structures of the core and tetramerization domains. The major tumor suppressor p53 is made of a disordered N-terminal transactivation domain (TAD), a disordered C-terminal regulatory domain, and two folded domains: a tetramerization domain separated by a linker from the core domain and a DNA binding domain, whose structures have been solved. Using BUNCH, a representative structure of full-length p53 could be reconstructed by modeling the backbone of all the unstructured regions absent in the crystal structures. Drastic conformational changes in the ternary and quaternary structure of p53 were observed between the full-length protein free in solution and the DNA-bound protein [84] (Fig. (**5**)) and even in a ternary complex involving DNA and the Taz2 domain of p300 bound to p53-TAD [85]. These unprecedented observations paved the way to a novel understanding of the mode of action of p53.

This approach might be considered somewhat restrictive because a single conformation cannot provide a comprehensive view of the ensemble of conformations that is explored by the object if it is flexible. Nevertheless, a single conformation may represent their conformational properties extremely well. For example, the structure of the full-length cellulase Cel48F, composed of a catalytic module tethered through a linker to a small dockerin domain, was modeled using CREDO [86], a precursor program of the more elaborate BUNCH. This program aimed to model missing regions in proteins whose crystal structures were incomplete because of flexible or disordered regions. Starting from the crystal structure of the catalytic domain, the program modeled the structure of the linker and of the dockerin region from the experimental scattering curve of the entire protein [77]. Several independent runs led to models that all exhibited a stretched region consistent with the number of residues of the linker following the catalytic domain and a small folded globular region, which was remarkably superimposable with the NMR structure of a homologous dockerin domain. These models differed from each other only by the orientation of the stretched region, suggesting fluctuating conformations of the linker region. In addition, all these models perfectly represented the experimental scattering curve. 

Modeling the conformation of the disordered region can be useful even when the rigid domains are very short. Models of the 70-residue long disordered salivary protein IB5 were constructed using BUNCH with only three short segments of polyproline repeats in the sequence modeled as rigid bodies. Each model, which was generated by 20 independent runs of BUNCH, displayed different conformations but with recurrent features (for example, extended conformations with large loops), and all were perfectly compatible with the experimental data [87].

This approach thus provides a single but highly relevant conformation, which is representative of the astronomical number of possibilities explored by the protein. Most of the time, this unique conformation describing the protein and its disordered region(s) is entirely sufficient and extremely valuable to answer the initial questions regarding the structural organization, the possible internal or external interactions with other domains or ligands, and the putative coordination or synergy of the different domains within the full-length protein or in complex. 

### 5.3 Comparison with High-Resolution Structural Models

While low-resolution models can be built using the modeling approaches described above, high-resolution structures or models of the protein or complex may be available or may have been built based on high-resolution data. Unlike many other biophysical techniques, one of the utmost advantages of SAXS lies in the possibility to calculate the theoretical scattering curve of a structural model and to compare it directly to the experimental data. 

Several programs have been developed, each of them essentially varying in their description of the hydration shell surrounding the protein, which affects the quality of the fitting at large q values. Up to q ≈0.3 Å^-1^, the different methods generally yield similar results. CRYSOL, developed by Svergun, has been the only available program for over a decade, and is still today the most widely used program. CRYSOL is moreover extremely fast and user friendly [88]. CRYSOL calculates the spherically averaged scattering curve with spherical harmonic multipole expansions. However, CRYSOL is limited by the number of spherical harmonics (maximum 50) and thereby by the size of the object of study. Therefore, when using CRYSOL for a protein with a large maximum dimension, which is typical for IDPs, the number of harmonics should be fixed at the maximum value, or another method should be considered. CRYSOL also assumes an implicit hydration layer of constant (but adjustable) density and fixed thickness. With improvements in instrumentation and the higher resolution now attained in the experimental scattering curves, most of the newly developed programs now consider explicit solvent in their calculation, leading to better fitting results, especially in the wide angle regime. This is often accompanied by a higher cost in calculation time, such as described for AXES (webserver: http://spin.niddk.nih.gov/bax/nmrserver/saxs1/) [89], and which depends on the algorithm selected to speed up calculations. While SASSIM uses multipole expansion [90], ORNL_SAS uses a Monte Carlo method (available at: http://www.ornl.gov/sci/csd/Research_areas/MS_csmb_comp_methods.htm) [91]. FoXS (webserver: http://modbase.compbio.ucsf.edu/foxs/about.html) uses the Debye formula to calculate intensities from atomic factors to which it adds a term that represents the displaced solvent and another term proportional to the solvent accessible surface to generate the contribution of the hydration water [92]. Other methods cleverly use a coarse-grain approach, taking advantage of the low-resolution of SAXS and significantly decreasing the computation time. Among them, the program Fast-SAXS (available at: http://thallium.bsd.uchicago.edu/ RouxLab/saxs.html) [93,94] proposes a more realistic description of the water shell based entirely on the atomistic description of water using molecular dynamics simulations. Another interesting approach has been proposed by Poitevin *et al.* [95] with the program AquaSAXS (webserver: http://lorentz.immstr.pasteur.fr/aquasaxs.php) in which the AquaSol method [96] is used to describe the hydration water as an assembly of self-orienting dipoles of variable density on a grid instead of a continuous dielectric medium. Finally, Stovgaard *et al.* [97] successfully reproduced protein scattering profiles even in the wide angle regime using coarse grained protein models and the Debye formula. However, their method did not describe the hydration layer surrounding the protein.

In the case of an IDP, it is still preferable to explicitly describe the water molecules surrounding the protein when comparing its theoretical scattering curve to the experimental data. Comparing the results obtained using several of these methods should confirm the best strategy to enhance the quality of the fit and to help refine and validate the atomic models, keeping in mind that interpreting the results of only SAXS data at the atomic level remains meaningless, considering the low-resolution of SAXS. 

These programs, which calculate the scattering curve from atomic models, may be particularly useful to confront experimental or modeled atomic structures to the structure in solution observed in SAXS. This was the case for the nuclear transcriptional activator protein TAT of the human immunodeficiency virus (HIV). TAT is an intrinsically disordered protein of ~100 residues and has long been at the center of antiviral therapeutic strategies because of its central role in viral replication. TAT is also a promising candidate antigen for anti-HIV vaccination. Several highly controversial structures of TAT from different strains of the virus have been solved by NMR, and the atomic coordinates have been deposited in the Protein Data Bank (PDB) [98-100]. On the other hand, a more recent thorough NMR study showed that the protein was highly disordered with no detectable residual structure or structural restraints and with characteristics similar to a random coil [101]. Using SAXS, it has been possible to test the validity of the structures deposited in the PDB by comparing their corresponding scattering profile to synchrotron scattering data [102]. These NMR structures were quite inconsistent compared with the experimental scattering profile (Fig. (**6**)) and with the dimensions inferred from the SAXS curve (R_g_, D_max_). Conversely, the SAXS data confirmed the study of Shojania and O’Neil [101] by showing that TAT was a disordered random coil [102]. 

Likewise, these programs can help build and validate models, as for the complex of the small intrinsically disordered thymosin-β4, which folds upon binding to G-actin and thus sequesters G-actin and regulates filament assembly [103]. Crystal structures of monomeric G-actin in the presence of inhibitors of polymerization could be obtained only with the N-terminal or C-terminal half of thymosin-β4. These crystal structures were combined to construct an atomic model of G-actin in complex with full-length thymosin-β4. The theoretical scattering profile of this model calculated using CRYSOL perfectly fit the experimental SAXS curve of this complex [103], supporting the validity of this model and the functional interpretation inferred from it.

Finally, as we will see below, these programs need to be used to calculate the scattering curves of the numerous atomic models that constitute the structural ensembles aiming to describe the distribution of conformations sampled by the protein in solution observed by SAXS. 

### 5.4 Distribution of Conformations

Intrinsically disordered proteins are clearly highly dynamic and do not exist as a single conformation in solution, but as interconverting conformers. Even when bound to a partner, they can still remain highly fluctuating, including on the interaction site, leading to what Tompa and Fuxreiter call fuzzy complexes [104]. 

Most spectroscopic techniques monitor the average signal arising from this multitude of conformations. A scattering pattern also contains the contribution of all these different conformations existing in solution. The approaches using SAXS described above often retrieve a single shape or conformation of the protein from this scattering pattern and do not describe the ensemble of the conformations. In some cases, it is even impossible to describe the scattering pattern with a single conformation. It is particularly striking when one examines the distance distribution functions of large, folded domains tethered by a long, flexible linker, as for the PCNA-Msh6-Msh2 complex (Fig. (**7**)) [74] or for the chimeric double cellulase Cel6AB, which was a pioneering case in which the distribution of conformations of an intrinsically disordered region was estimated using SAXS [105]. In such cases, it is interesting to find the right distribution of conformations that agrees with the experimental data. Furthermore, when structural and dynamic information from other complementary techniques are available and can be combined with SAXS data, it may be worth trying to gain further insights into the dynamics of the flexible regions of the IDP and to establish the ensemble of populations existing in solution, particularly in the case of multidomain proteins containing disordered regions, whatever their length. Deciphering the ensemble of conformers that the protein can reach is crucial as this would allow a comprehensive understanding of the energy landscape explored by the disordered proteins and possible insights on some of the conformers that significantly differ from the average or more stable conformation but that may play a critical role in the function of the protein. 

Retrieving the ensemble of conformations adopted by a protein from experimental data is quite challenging. The number of degrees of freedom is very large compared to the constraints provided by the experimental data, which inexorably leads to a degenerate solution. As a consequence, it is not possible to obtain a unique solution, and on the contrary, many different ensembles may be consistent with the data. This is even more acute for SAXS, which is already an underdetermined technique. Overfitting the data is thereby a serious pitfall that one must try to avoid by all possible means [106].

Several experimental approaches, including NMR (PREs, RDCs, NOEs), FRET, and SAXS, have been used to build ensembles of structures that describe the dynamic properties of IDPs [107, 108]. Reviewing all these methods is beyond the scope of the present review, and we will focus here only on those techniques that use SAXS data, either exclusively or in combination with other experimental measurements. 

The strategy to establish a distribution of conformations in accordance with experimental SAXS data can be described by a general scheme in three main steps, each step having its own specific difficulties: (i) generating a comprehensive library of conformers, (ii) calculating the theoretical scattering profile of each of these conformers, and (iii) selecting a subset of these conformers whose scattering curve of the ensemble best fits the data. 

The first step is not specific to SAXS. In particular, the recent advances in NMR, including RDCs and PREs, have urged the development of programs that generate wide pool of structures to reproduce biophysical data obtained on highly dynamic macromolecules [106, 108]. The main difficulty here is to generate a broad enough pool of conformers in a reasonable computing time. Molecular dynamics (MD) generates numerous atomic structures along a trajectory with adequate force fields. However, depending on the size of the protein, MD may not sample a sufficiently wide library of conformers in a reasonable computing time, considering the large conformational space explored by intrinsically disordered proteins. Several strategies have therefore been used to circumvent this difficulty. A typical workaround is found in the program Flexible-Meccano, which generates coarse-grained realistic atomic models using a Monte-Carlo technique and applies backbone dihedral angles allowed in the Ramachandran space [109]. Approaches using this program have been successfully applied to many IDPs to reproduce SAXS or RDC data [110-114]. 

The difficulties of the second step concern the accuracy of calculating a theoretical scattering curve of models by estimating the correct contribution of the hydration layer, as discussed in the paragraph above. The third and most critical step faces the redundancy of the possible solutions in selecting several structures whose average signal fits the data, and safeguards have to be defined to prevent overfitting. 

Several program suites have been developed that integrate the three above-described steps to generate structural ensembles compatible with the SAXS data. These programs adopt different strategies at each step, especially concerning the choices made to minimize overfitting. 

The program suite EOM, Ensemble Optimization Method [115], is currently the most popular due to its simple interface. It is widely used when an IDP is examined using SAXS. The first step of the procedure is performed by the program RanCh (Random Chain), which builds a pool of random models of IDPs or multidomain proteins with linkers from the sequence of the full-length protein and the atomic coordinates of the folded domains (if any). The disordered regions are modeled with C_α_ chains using a quasi-Ramachandran plot. The authors recommend generating 10,000 structures. The theoretical scattering profile of all these structures is then calculated using CRYSOL. The third program, Gajoe, (Genetic Algorithm Judging Optimization of Ensembles) uses a genetic algorithm to select an ensemble of scattering curves (and thereby of structures) whose average fits the data. Typically, several dozens of individual scattering curves are selected. The results are presented as a histogram of the radii of gyration and of the D_max_ of the selected protein models compared to the distribution of R_g_ and of D_max_ of the initial random pool. Instead of using RanCh, the user can start from a pool of conformers generated by any other method, thereby adding restraints arising from other experimental results, such as PREs. The pdb files of the models of the ensemble that give the best fit are also provided. These models do not necessarily represent the structures adopted by the protein but are just models whose average calculated scattering curves best fit the data. In the case of an entirely disordered protein, EOM provides an estimate of the conformational landscape and of the shift in the dimensions of the ensemble of conformations reached by the protein with respect to those of a random coil, similar to when one compares the dimensions and structural parameters inferred from the experimental curves (R_g_, D_max_, statistical and contour lengths) to those of a random coil. EOM thus provides an alternate way to reveal structural restraints along the polypeptide chain at the global scale. In the case of multidomain proteins, EOM can provide information on the flexibility of the interdomain linkers, comparing them to a random distribution or with variant linkers [116, 117] and is particularly productive when coupled with NMR data [45]. The fluctuations of the linkers in the multidomain ribosomal L12 protein were thus investigated, and an ensemble model of the structure and reorientational dynamics of the protein were obtained by reconciling SAXS data with NMR relaxation data, which enabled a detailed description of the structural propensities of the linkers [118]. In some cases, bimodal distributions may be yielded by EOM calculations [119-121] and may provide interesting insights into a possible equilibrium between different preferred populations. A prudent interpretation of the results at the functional level is recommended here, and such results would highly benefit from being consolidated by other biophysical techniques, such as FRET for example. 

The recent program Broad Ensemble Generator with Re-weighting (BEGR), initially developed to interpret NMR chemical shifts of proteins, appears promising because it generates realistic structural ensembles in a broader conformational space, by applying only steric constraints. The probability (*i.e*., weight) for each structure in the pool is determined such that the average simulated spectrum best fits the experimental spectrum using a Metropolis Monte Carlo approach [122].

The program suite SASSIE [123] has been originally written to generate a set of structures for the HIV Gag protein consistent with SANS data and neutron reflectivity data. SASSIE is executed from within the Visual Molecular Dynamics (VMD) program [124] and utilizes molecular dynamics with CHARMM force-fields to generate large ensembles of structures by randomly varying backbone dihedral angles with energetically allowable values. Distance constraints such as those provided by NOEs or other techniques may be applied when generating these structures. Each structure is then energy minimized using the program NAMD [125]. The theoretical scattering curve of these structures is calculated using CRYSON [126] or Xtal2Sas [127] for SANS, and CRYSOL [88] for SAXS. The scattering curves are then analyzed and compared to the experimental profile through the χ^2^ (discrepancy between the theoretical and the experimental profiles) and the radius of gyration. No particular weighting scheme is applied, so that a single structure or a linear combination of several structures may be selected as the best representative structures reproducing the experimental data. 

Several other approaches have recently been developed with a different philosophy. All of the following approaches try to prevent overfitting of the data by selecting an ensemble of the minimal size that best fits the data. Most of them also use further restraints or strategies to strengthen and assess the validity and robustness of the solution.

The program Minimal Ensemble Search, MES (freely available for academic use at http://bl1231.als.lbl.gov/saxs_protocols/mes.php) aims to determine the minimal ensemble that best fits the data [27]. A range of random structures is generated by the program BILBOMD, which combines MD at a high temperature to avoid local minimum trapping with rigid body modeling of the globular domains. Distance constraints can also be added. This approach shares some similarities with the constraints solution scattering modeling method developed by Stephen Perkins [128, 129], apart from the fact that the latter tends to select only one best-fit conformer whose atomic coordinates are then deposited in the protein Data Bank. The theoretical scattering curves of the models yielded by BILBOMD are then computed using CRYSOL, and the selection of the minimal ensemble is performed by a Monte Carlo genetic algorithm. Restraints to limit overfitting are provided by realistic conformational models explored by MD and by the selection of a minimum of structures that deconvolute the SAXS data, usually two to five weighted conformations. The flexibility of the protein is assessed by comparing the root mean square deviation (rmsd), R_g_ and D_max_ between the selected models and the best-fit model. 

The Basis-Set Supported SAXS (BSS-SAXS) reconstruction is also an integrative approach [28]. Simulations using coarse-grained molecular dynamics with different initial conditions and incorporating constraints, such as interactions between different domains of the proteins, adjusted to match the Kd are first performed to ensure a proper sampling of the accessible conformational space. The theoretical scattering curves of the different models are calculated using Fast-SAXS, as described above. A Bayesian-based Monte-Carlo procedure yields fractional populations with more accurate statistics. The originality of the approach also lies in the selection not of a limited number of discrete conformers, but of a limited number of representative families of states that actually cluster around a large ensemble of configurations (Fig. (**8**)). This approach elucidated the assembly conformational states of the multidomain protein Hck, from the family of Src-kinases. Importantly, it also revealed the dynamic equilibrium between several closed inactive and open active conformations regulated by the interaction forces between the different domains (SH3, SH2 and linkers) and how this equilibrium is perturbed and shifted towards a family of conformations upon binding to different signaling peptides. These results provided a critical understanding of the mechanisms of regulation of this family of kinases, which is involved in many vital and cancer-related signaling pathways [28, 130]. 

Another Ensemble-Refinement of the SAXS (EROS) method has recently been developed to determine the dynamic conformational properties of biomolecular assemblies containing intrinsically disordered segments [30]. An initial ensemble of conformations is first generated using coarse-grained models, which are then elegantly refined by an energy function optimized for protein binding in which the interactions between domains are treated at the residue level with appropriate energy potentials, such as electrostatic potentials or hydrophobic interactions. The theoretical scattering curves of the models are calculated using an algorithm that is similar to CRYSOL but adapted to coarse-grained and not atomic models. Then a maximum entropy refinement selects the minimum weighted clusters of structures that are consistent with the SAXS data to prevent overfitting. As the generated models account for hydrophobic and electrostatic interactions, drastic conformational reorganizations upon increased salt concentrations of the endosome associated CHMP3, a key component of the ESCRT-III complex, could be described in detail. Electrostatic interactions between domains, which ensure an auto-inhibited compact closed conformation of CHMP3, were disrupted when shielded by high salt concentrations, leading to the active open and extended conformations, with a higher flexibility. Indeed, a minimum of 60 structures was required to account for the SAXS data of the open conformation at high salt concentrations, whereas an ensemble of only 6 clustered structures agreed with the SAXS data at low salt concentrations [30, 31]. Similarly, the equilibrium between the open and closed conformations of the heterotetramer complex ESCRT-I was deciphered using the same approach, and an ensemble of six structures was required to fit the scattering data coupled to double electron-electron resonance spectroscopy of spin-labeled complexes and confirmed by FRET spectroscopy measurements [131]. 

The program ENSEMBLE was originally written to describe the ensemble of populations of a folded and unfolded N-terminal SH3 domain of drk co-existing under non-denaturing conditions [132]. Since then, it has been further developed [32, 133] and has been used for several intrinsically disordered proteins [134-136]. ENSEMBLE utilizes several strategies to prevent overfitting. First, the program can account for a high number of restraints from many different experiments, such as NMR chemical shifts, NOEs, J coupling constants, RDCs, PREs, tryptophan indole fluorescence, hydrodynamic radius, and SAXS. Second, the minimum ensemble of structures compatible with all the data is selected to represent the conformational space explored by the protein and the variety of conformations that may be attained by the protein. ENSEMBLE first employs the program TraDES [137] to generate a wide range of statistically random structures, taking into account the secondary structure propensities if necessary. For the ensemble minimization procedure, experimental results from the various methods are converted into energy values, distances restraints, or solvent-accessible areas, depending on the information provided by the technique. Theoretical scattering data of the generated models are computed using CRYSOL. ENSEMBLE can thus be used to observe significant transient structures in the free disordered protein Sic1 and to describe the highly dynamic interactions between the protein and the Cdc4 subunit of a ubiquitin ligase together with the role of phosphorylation of Sic1 in interchangeable interactions [134, 138]. Forman-Kay’s group also investigated the structural propensities of several intrinsically disordered regulators of the protein phosphatase 1 (PP1) in the unbound state and in complex with the inhibitor-2 (I-2) [135]. Among the ensemble of selected structures (~10-20 structures) in the free state compatible with all the experimental restraints (chemical shifts, PREs, R_h_, SAXS), transient secondary structure elements were observed; also, preformed structural motifs similar to those in the bound state were present with a sufficient stability to facilitate the interaction with PP1. This result thereby supports the longstanding idea that the selection of a prefolded conformer with pre-structured Motifs (PreSMos, see [139] in the same CPPS issue, for a review) could be the predominant model for certain interactions, besides the folding upon binding mechanisms observed by Sugase *et al. *[140]. Finally, the ensemble of dynamic structures of the complex of PP1 and I-2 generated using ENSEMBLE was examined. Consistent with the scattering curve of the complex, these data revealed that I-2 remains largely disordered upon complex formation. Nevertheless, transient contacts were identified that were not observed in the partial crystal structure of the complex, providing the first molecular insights into the function of a region of I-2 that plays a critical biological role in the interaction with PP1 [135].

Determining the ensemble of conformations of a disordered protein from SAXS data is therefore quite a challenge and requires clever, rigorous procedures and cross-validation with a maximum of complementary techniques to decrease the redundancy of the solution. The information yielded by the obtained models is however of crucial interest because it provides unique information on the conformational dynamics of IDPs, although not on the motional timescales, and on transient or local structures that may be critical for the activity of the protein. A minimum number of representative models on the distribution of conformations prevents overinterpreting the models. The authors all discuss their results and agree on the fact that the selected conformers do not represent the only conformations nor the most stable conformations attained in solution. Instead, the selected structures are rather a snapshot of a large continuum of states. The global properties of the models and their recurrent features are captured in these representative conformers and may provide information on the presence of local rigidities, transient structures, or accessible conformational states that may be functionally relevant. These refined ensembles increase the resolution of SAXS beyond a simple overall shape or average global conformation and can provide submolecular detailed information even at atomic resolution provided that high-resolution data are available. A plethora of information on the biological activity of intrinsically disordered proteins can hence be inferred at the molecular level and can be extremely valuable to deciphering their role in the cell. 

## 6 PROTEIN INTERACTIONS AND SANS

During the last decade, SAXS has become increasingly successful in the study of biological macromolecules in solution. Because of its tremendous potential in the study of macromolecular complexes using the contrast variation method, small angle neutron scattering (SANS) is extremely promising for the study of IDPs. Essentially, SANS provides the same kind of information as SAXS, as described above. The difference lies in the fact that X-rays interact with electrons whereas neutrons interact with nuclei, allowing the possibility to use isotopic labeling, especially hydrogen and deuterium, which exhibit very different scattering lengths. By varying the D_2_O/H_2_O content of the solution, an object can be rendered invisible with a scattering density identical to the solvent, at the given D_2_O/H_2_O matching point. This matching point differs for DNA, RNA, lipids, polysaccharides, proteins, and perdeuterated proteins. It is therefore possible to focus only on part of a macromolecule or complex by matching the rest of the object at the corresponding D_2_O/ H_2_O matching point. 

To our knowledge, very few studies have utilized SANS to analyze IDPs. The mammalian translation elongation factor 1A (eEF1A) was shown to be significantly disordered using SANS [141]. Similarly, the structural conformation of HIV-Gag, which is composed of several globular and coil domains, was elucidated using SANS [142]. In these two examples, SANS was employed in exactly the same way as SAXS would have been employed. More recently, the conformational changes of HIV-Gag upon binding to a small nucleic acid were investigated by SANS at different D_2_O/ H_2_O contrasts [143]. Furthermore, a recent review has illustrated the combined use of NMR, SAXS, and SANS using the contrast variation method to build a model of the tandem RNA recognition motif domains (RRM1 and RRM2) of the human splicing factor U2AF65 bound to an oligonucleotide in which the flexibility of the linker tethering the RRM1 and RRM2 domains was described using a large ensemble of conformations consistent with the RDC data and the X-ray and neutron scattering curves [144].

A new milestone has been reached with the recent study of Johansen *et al.* [145] in which SANS with contrast matching was used to investigate the effect of macromolecular crowding on the conformation of an IDP. They mixed perdeuterated N protein of bacteriophage lambda, a small intrinsically disordered protein, with the small hydrogenated bovine pancreatic trypsin inhibitor (BPTI) as a crowding agent at increasing concentrations up to 130 mg/mL at the matching point of 42% H_2_O, at which BPTI becomes invisible. Their results tend to indicate a compaction of the disordered protein at relatively low macromolecular crowding, as was observed for random coil polymers in crowded conditions [146, 147], but this effect apparently does not increase in denser crowding conditions. This study provides crucial answers to the recurrent questions about the behavior of IDPs in the crowded cell, which is likely to be completely different than in the relatively dilute test tube. Furthermore, the use of contrast variation SANS between two different proteins involving an IDP promises new exciting advances in the characterization of conformational changes in disordered proteins occurring between the free and bound states with an unlabeled protein partner. 

## 7 CONCLUSION

IDPs are particularly recalcitrant in structural studies, which has long hampered their structural characterization. SAXS and SANS are now widely recognized as indispensable tools for analyzing these proteins. Since the introduction of the protein trinity concept [148,149], IDPs have been associated with the random coil state of denatured proteins and were mostly considered as devoid of any significant structural features. Lessons from recent SAXS studies, especially those coupled with NMR data, revealed that random coil-like IDPs are significantly different than the random coil of the denatured state. Upon denaturation, all of the interaction potentials between the residues of the polypeptide chain, which stabilize the scaffold of the native protein, are strongly altered or screened because of the denaturing conditions, leading to unfolding and a random coil set of conformations. In the case of intrinsically disordered proteins, the residues specificities in the sequence are not screened by any denaturing condition. Although their strong sequence bias prevents them from folding onto a hydrophobic core and causes them to maximally expose the polypeptide chain to the solvent [150], residual local and even long-range weak interactions may still occur. These transient or sometimes more stable elements of structure are likely to be the ones that play crucial roles in the function of IDPs, such as in the recognition process. The most recent advances in SAXS and SANS in the instrumentation, methodology, and computational analysis of the data enable one to extract all or almost all of the information content of the scattering profile of an IDP likely to describe these important features. Whatever the degree of disorder of an IDP, from fully disordered proteins to multidomain proteins with only short disordered segments linking globular domains, SAXS can describe the conformational space explored by the protein, decipher the functional structural organization of multidomain complexes, detect subtle rigidities important for the function along the polypeptide chain, and provide a wealth of other information on the structural properties of IDPs. Finally, the immense capacity of SAXS to decipher the structural features of IDPs, with or without globular domains, is fully attained when it is used with other biophysical and computational techniques, which allow one to access a plethora of information and thereby to describe the protein as a whole across structural scales. With the possibility offered by these integrated approaches at low and high resolutions to examine an ensemble of generated structures that reconcile all of the experimental data, the existence of minority conformers that may be critical for the function of the protein, preformed structural elements likely to be binding motifs, or any structural features crucial for the biological activity might hence be observed. The examples described above illustrate how crucial and fundamental questions on the function of IDPs can be addressed and reveal insights into their unique structure-function relationships. The present state of the art of the SAXS approaches together with future developments in SAXS aiming to integrate information from more and more complementary techniques, as well as in SANS with the first examples discussed above, open new avenues towards a comprehensive understanding of the structural and biological activity of these proteins with particularly unique properties. 

## Figures and Tables

**Fig. (1):**
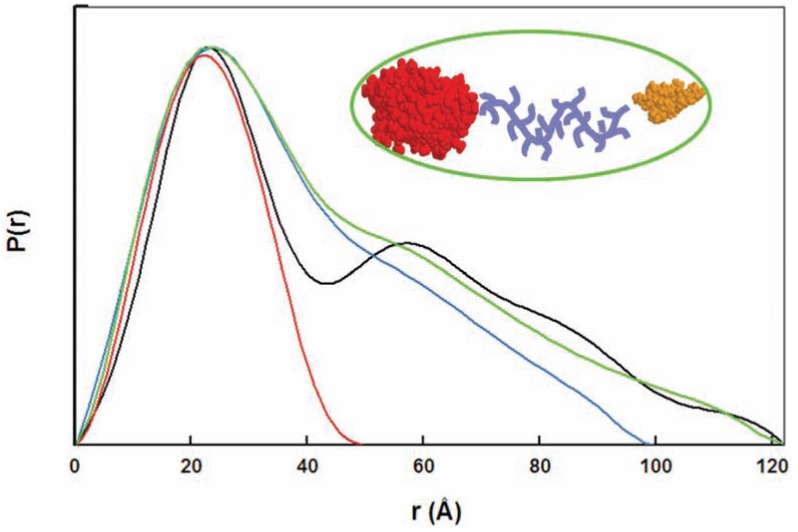
Experimental P(r) functions of multidomain proteins. Experimental P(r) functions of the *Humicola insolens* cellulase Cel45 and variants: globular catalytic domain (red curve), catalytic domain and linker (blue curve), full-length Cel45 wild-type (green curve), and full-length Cel45 with a proline mutation leading to a more rigid linker (black curve). The crystal structures of the catalytic domain (red) and the cellulose-binding domain (yellow) are represented in space-filling mode. The enhanced rigidity of the linker in the mutant Cel45 translates into a P(r) function with a well separated peak corresponding to the interdomain distances. (Figure adapted from [60]).

**Fig. (2):**
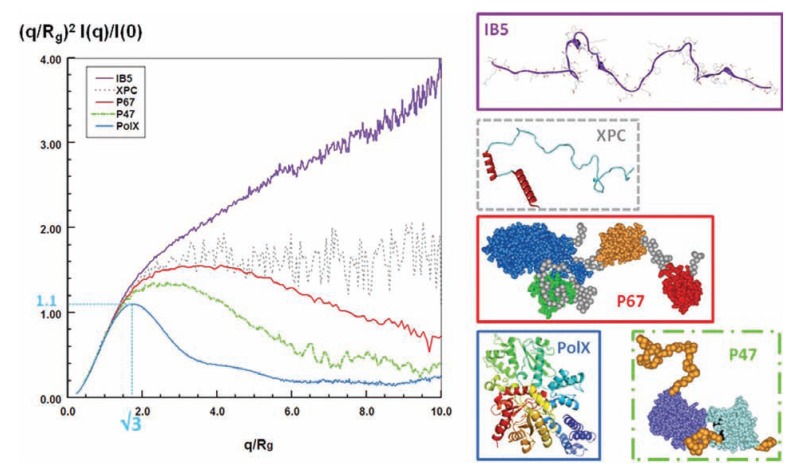
Normalized Kratky plots. The scattering pattern of globular proteins in a normalized Kratky plot exhibits a bell-shaped profile with a clear maximum value of 1.104 for qR_g_=√3, regardless of the size of the protein, and are all nearly superimposable in the q range 0<qR_g_<3. Conversely, for a random chain, the curve rises with increasing angle, to nearly reach a plateau between 1.5 and 2 and may further increase at q>0.2-0.3 Å^-1^, depending on the persistence length and the internal structure of the protein. Bell-shaped profile of a globular protein (PolX, blue line); curve of a protein consisting of several domains tethered by linkers with rather compact conformations (p47^phox^, dotted green line) or extended conformations (p67^phox^, continue red line); curve of a fully disordered protein with very short elements of secondary structure (XPC dotted grey line); and curve of a fully disordered and extended protein with short segments of polyproline repeats (salivary protein IB5, continue purple line).

**Fig. (3):**
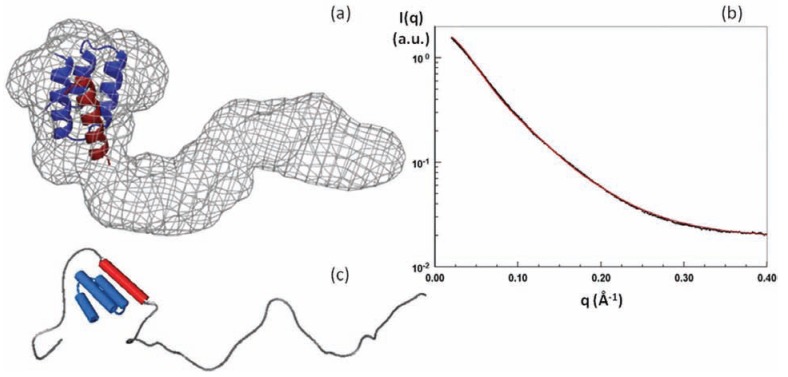
**3D model of the complex N_TAIL_-XD of the measles virus.** (**a**) The envelope of the complex calculated by GASBOR exhibits a bulge that was recurrent from run-to-run calculations and can accommodate the crystal structure of XD (blue) and the alpha-MoRE of N_TAIL_ (red). The elongated region of the envelope was more variable in shape upon several calculations with GASBOR. (**b**) The scattering curve of the envelope calculated with GASBOR (red curve) perfectly fits the experimental scattering curve of the complex (black curve). (**c**) A molecular model of the full-length complex was also obtained from the SAXS data using CREDO, which reconstructed the missing disordered region of the crystal structure of the complex (Figure adapted from [78]).

**Fig. (4):**
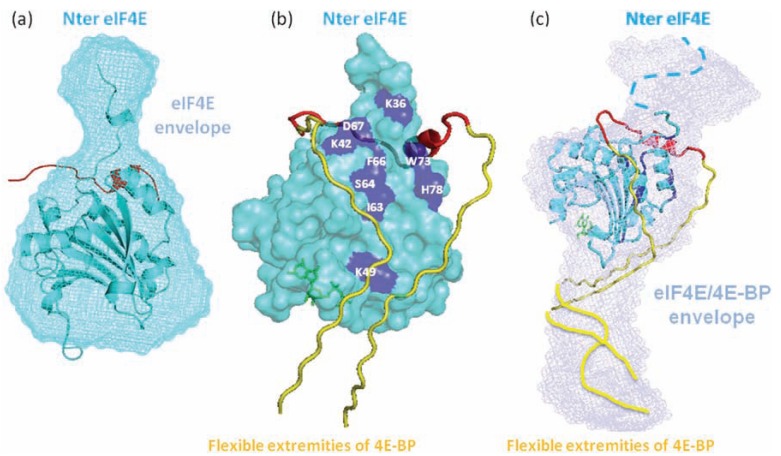
**Shape calculation and 3D model of the complex eIF4E bound to 4E-BP.** (**a**) The crystal structure of 4E-BP (cyan) is perfectly superimposable on the envelope of the free protein in solution calculated with DAMMIN from the scattering data, with a small bulge corresponding to the disordered N-terminus of 4E-BP. (**b**) X-ray crystallization showed that a short region of eIF4E (red) visible in the electronic density undergoes an induced folding into an alpha-helix upon binding to 4E-BP. NMR studies identified other residues of 4E-BP involved in the interaction (dark blue) on the opposite side of the protein where the alpha-helix of eIF4E binds 4E-BP, suggesting that eIF4E (yellow) wraps around 4E-BP but retains enough flexibility to not be seen by X-ray crystallography. (**c**) The envelope of the complex calculated with DAMMIN exhibits an upper-half region identical to the shape of 4E-BP alone and an elongated region on one side of 4E-BP, probably corresponding to the disordered portion of eIF4E. This envelope, together with data from X-ray crystallography and NMR, allowed the authors to propose a model of the complex with a rather well defined region in the close vicinity of 4E-BP and a loose region corresponding to the rest of eIF4E that remains mostly disordered in the complex (yellow). (Figure adapted from [76]).

**Fig. (5):**
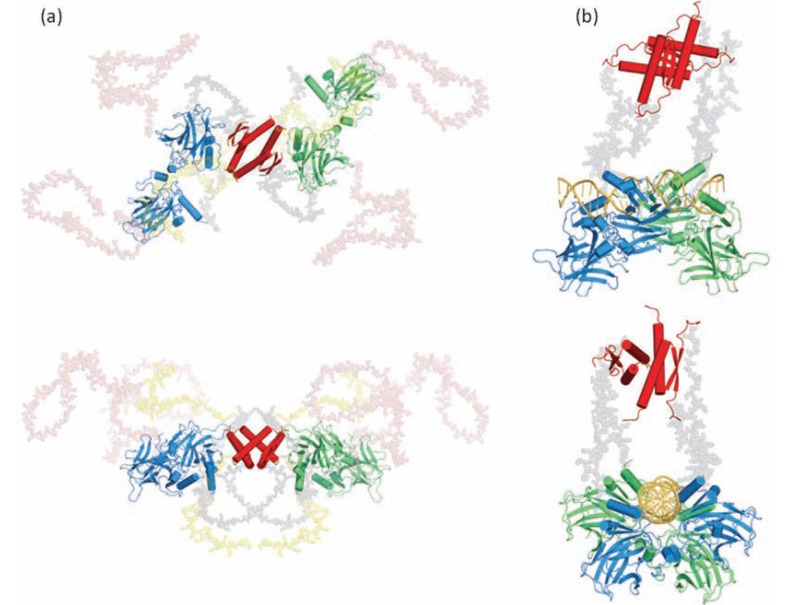
**3D models of p53.** SAXS models of free (**a**) and DNA-bound (**b**) p53 in solution from rigid body analysis with the addition of missing fragments by BUNCH. Both models are shown in two orthogonal orientations. Core (green and blue) and tetramerization (red) domains are shown as cartoon representations, with core domains binding to the half-site in the same color. Flexible connecting linkers (gray), N termini (pink), and C termini (yellow) are shown as semitransparent space-filled models. Models of the flexible regions are approximations to illustrate their global structural properties rather than representing defined conformations. (Figure taken from [151]).

**Fig. (6):**
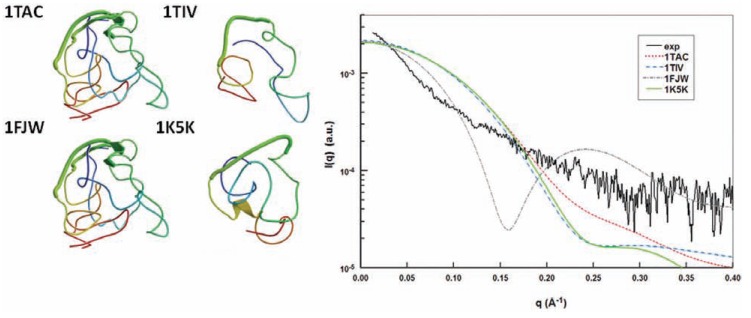
**Use of CRYSOL to compare atomic structure with the structure in solution observed by SAXS.** Comparison of experimental SAXS data from HIV-TAT (black line) with the theoretical scattering curve of published structures of TAT with pdb code 1TAC (red dotted line), 1TIV (blue dashed line), 1FJW (grey dash-point line), and 1K5K (continue green line)] using the CRYSOL program to show the discrepancy between the pdb structures and the structure in solution observed by SAXS. The low statistics of the experimental curve are accounted for by the low concentration of the protein (< 1 mg/mL) (Figure adapted from [102]).

**Fig. (7):**
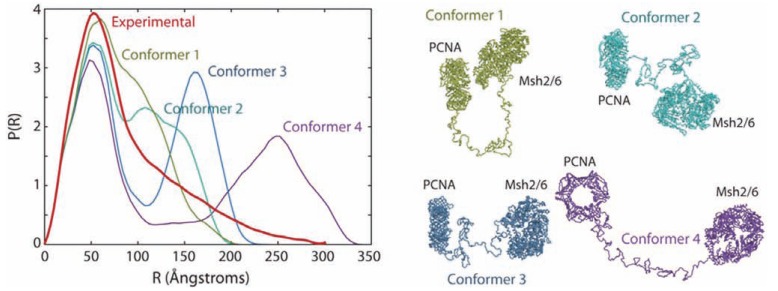
**Comparison of experimental and calculated distance distribution functions of flexible multidomain proteins.** P(r) functions calculated for four randomly generated models of Msh2-Msh6 linked to PCNA via random peptides with different interdomain distances reveals that no single conformer can account for the observed P(r) curve of the Msh2-Msh6-PCNA complex. The red curve with the long tail corresponds to the experimental P(r) curve of Msh2-Msh6-PCNA complex. (Figure adapted from [74]).

**Fig. (8):**
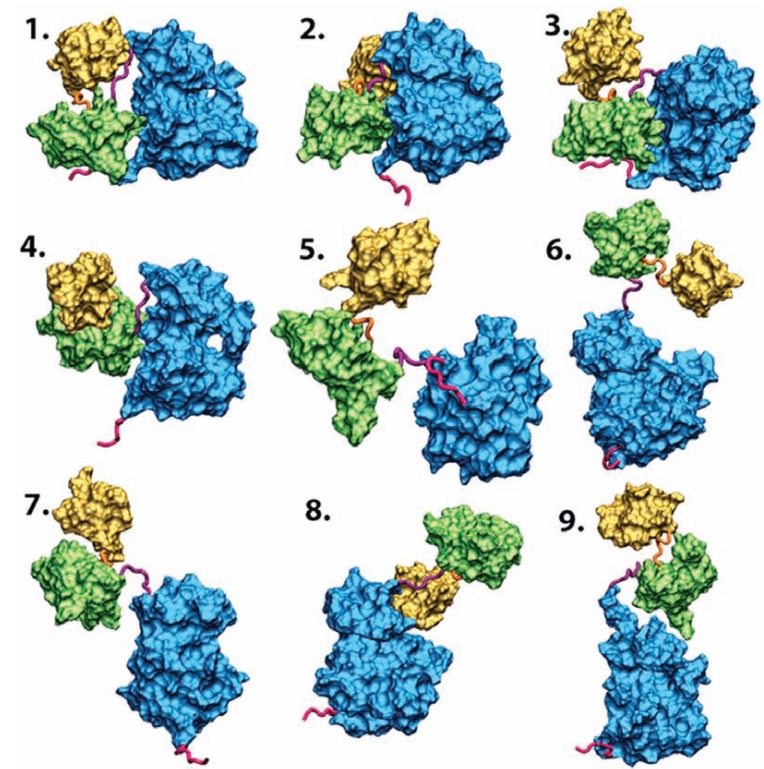
**Representatives of the 9 families of conformational states of the Src-kinase Hck.** The program BSS-SAXS first generated numerous conformations and clustered them into 9 representative families of states ranging in architecture from fully to partially assembled and disassembled states, in size from compact to extended forms, and in interdomain separation from fully assembled to partially disassembled states. The catalytic domain is in blue, the SH2 domain is in green, the SH3 domain is in yellow and the linkers are in red. Based on the experimental scattering data of free Hck in solution, Hck exists in different open and closed conformations stabilized by intramolecular interactions involving the SH2 and the SH3 domains. The assembled conformation state 1 is the major species (83%) and is in equilibrium with minor states 6 and 8. In the presence of two activating peptides, the scattering data indicate that only the open conformations corresponding to states 5 and 6 exist in solution. (Figure taken from [28]).

## ABBREVIATIONS

CD: = Circular Dichroism
DLS: = Dynamic Light Scattering
FRET: = Förster/Fluorescence Resonance Energy Transfer
IDP: = Intrinsically Disordered Proteins
IDR: = Intrinsically Disordered Region
MD: = Molecular Dynamics
NMR: = Nuclear Magnetic Resonance
NOE: = Nuclear Overhauser Effect
PFG-NMR: = Pulse-Field Gradient NMR
PRE: = Paramagnetic Relaxation Enhancement
RDC: = Residual Dipolar Coupling
rmsd: = root mean square deviation
SAS: = Small Angle Scattering of X-rays or neutrons
SAXS: = Small Angle X-ray Scattering
SANS: = Small Angle Neutron Scattering
